# Intimate partner violence among HIV positive women in care - results from a national survey, Uganda 2016

**DOI:** 10.1186/s12905-019-0831-1

**Published:** 2019-11-01

**Authors:** Steven Ndugwa Kabwama, Justine Bukenya, Joseph K. B. Matovu, Violet Gwokyalya, Fredrick Makumbi, Jolly Beyeza-Kashesya, Shaban Mugerwa, John Baptist Bwanika, Rhoda K. Wanyenze

**Affiliations:** 1 Makerere University School of Public Health, Kampala, Uganda; 2 Department of Community Health and Behavioral Sciences, Makerere University School of Public Health, Kampala, Uganda; 3 Department of Disease Control and Environmental Health, Makerere University School of Public Health, Kampala, Uganda; 4 Department of Epidemiology and Biostatistics, Makerere University School of Public Health, Kampala, Uganda; 5 Mulago National Referral Hospital, Kampala, Uganda; 6 AIDS Control Program, Ministry of Health, Kampala, Uganda

**Keywords:** Intimate partner violence, HIV positive women, Uganda

## Abstract

**Background:**

Women remain disproportionally affected by the HIV/ AIDS epidemic because of sociocultural factors including violence perpetrated by intimate partners. Among HIVpositive (HIV+) women, intimate partner violence (IPV) affects engagement in care and reproductive health outcomes. We analyzed data from a national survey to estimate the prevalence of IPV among HIV+ women in care and associated factors.

**Methods:**

The study was conducted among 5198 HIV+ women in care. Data were collected on socio-demographic characteristics, self-reported couple HIV status, mutual HIV status disclosure and IPV. IPV was assessed by asking participants whether their current husband or partner ever hit, slapped, kicked or did anything to hurt them physically, and whether their current husband or partner ever physically forced them to have intercourse or perform any sexual acts against their will. Women who responded “yes” were classified as having ever experienced IPV. Modified Poisson regression was used to identify factors associated with experiencing IPV.

**Results:**

Of 5198 HIV+ women, 1664 (32.1%) had ever experienced physical violence, 1466 (28.3%) had ever experienced sexual violence and 2290 (44.2%) had ever experienced any IPV. Compared with women in relationships where the woman and their male partner were of the same age, women in relationships where the partner was ≥1 year younger were more likely to ever experience IPV (Prevalence risk ratio [PRR] = 1.43, 95% Confidence Interval [95%CI]: 1.10–1.71), as were women in relationships where the partner was < 10 years older (PRR = 1.20, 95%CI: 1.00–1.43) or ≥ 10 years older (PRR = 1.31, 95%CI: 1.05–1.64). Compared with women who did not have biological children, women with 3–4 biological children were more likely to have ever experienced IPV (PRR = 1.27 95%CI: 1.00–1.59) as were those with ≥5 biological children (PRR = 1.34, 95%CI: 1.06–1.71). Compared with women in sero-concordant relationships, women in sero-discordant relationships were less likely to ever experience IPV (PRR = 0.87 95%CI: 0.78–0.98).

**Conclusions:**

In Uganda, a high proportion of HIV+ women have ever experienced IPV. Experiencing IPV was associated with circumstances related to the intimate relationship between the woman and her male partner. Health care workers should screen HIV+ women in care for IPV and offer appropriate psychosocial assistance.

## Background

Data from Demographic Health Surveys of 18 countries across Sub-Saharan Africa have shown that women are disproportionally affected by the HIV/ AIDS epidemic compared with their male counterparts [1]. The gender disparities in the HIV/ AIDS burden have been attributed to biological factors such as vaginal microbiology and ecology, and socio-demographic and behavioral factors such as a divergence in the risk for HIV infection including age at sexual debut, poverty and violence [2, 3]. Violence in all its forms can significantly inflate the risk of HIV infection particularly in communities that are traditionally patriarchal and where rape myths and violence against women perpetrated by an intimate partner are endorsed by both women and men [4]. The 2016 Uganda Demographic Health survey revealed that 49% of women and 41% of men agreed with at least one justification for physical violence by men against their female partner [5]. The World Health Organization (WHO) defines intimate partner violence as any behavior in an intimate relationship that causes physical, sexual, or psychological harm to those in the relationship [6]. This behavior can be categorized as physical violence including hitting, slapping, kicking or beating; sexual violence involving sexual coercion and forced sexual intercourse; psychological/ emotional abuse including belittling, insults, intimidation, humiliation and threats of harm while controlling behavior entails isolating a person from family and friends and restricting access to financial resources [7]. Although there are reports of men suffering different forms of violence inflicted by their female intimate partners, the violence perpetrated by men overwhelmingly outstrips that inflicted by women [8, 9]. Intimate partner violence against women occurs in all societies irrespective of social, economic, religious, cultural or ethnic variations with 10–69% of women across 48 countries globally reporting being physically assaulted by an intimate male partner [6]. Among women who are HIV positive, having a partner that is physically, emotionally or sexually abusive can affect their engagement in HIV care and treatment. A systematic review and meta-analysis showed that intimate partner violence was significantly associated with lower anti-retroviral therapy (ART) use, lower self-reported ART adherence and lower odds of viral suppression [10]. Physical, emotional or sexual abuse in a relationship is likely to preclude the perceived satisfaction with partner support which has been associated with ART adherence [11]. Furthermore, intimate partner violence has been associated with a loss of reproductive health control that may involve coercion by the male partner for the female partner to become pregnant and birth control sabotage or male partner interference with contraception [12]. This could lead to a woman becoming pregnant against their will, increase risk of sexually transmitted infections or among women who are HIV positive in a sero-discordant relationship increase the risk of HIV transmission to their partner.

In 2016, a national health facility-based survey was carried out in Uganda to assess the uptake of family planning services and establish the unmet need for family planning services among HIV infected women in care. Participants were also asked about their experience of physical and sexual violence perpetrated by their partner. We analyzed data from this survey to establish the prevalence of intimate partner violence against women in HIV care and assess the factors associated with experiencing intimate partner violence. Findings from the analysis will inform the integration of strategies to prevent and control intimate partner violence into HIV care and treatment programs for women living with HIV.

## Methods

**Fig. 1:**
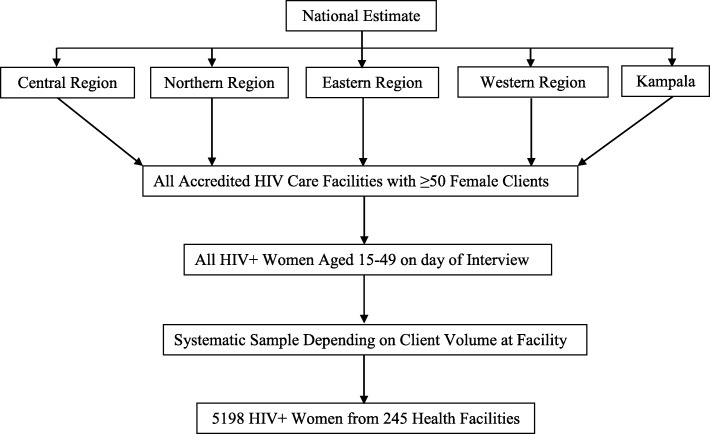
Flow chart of selection of HIV+ women to participate in the survey

This was a secondary analysis of data from a facility based cross-sectional study that was conducted across the 5 geographical regions of Uganda to assess the uptake of family planning services and establish the unmet need for family planning services among HIV infected women in care. A detailed description of the methods including the study sites, sample size determination and sampling procedures have been published elsewhere [13]. Briefly, the sample size was calculated assuming *p* = 30% unmet need for family planning HIV+ women in care, 3.6% margin of error, 5% type I error rate, design effect = 1.5 and a non-response rate of 10% giving an overall sample size of 5185. Since the assessment of intimate partner violence was a secondary objective from the survey, considering a total population of about 600,000 HIV positive women in Uganda [14] and a background prevalence of spousal violence of 44% [5], a minimum sample size of 5185 would provide sufficient power to establish the national prevalence of intimate partner violence among HIV positive women. The sampling was done in two stages. First, an equal number of accredited HIV care facilities with at least 50 female clients were randomly selected from a sampling frame of accredited HIV care facilities in Uganda (Fig. 1). The next stage of sampling was at facility level where by all women aged 15–49 years who presented for HIV care at the facilities on the interview days were listed on attendance forms. We then used systematic sampling to select the number of eligible women using a sampling interval based on the client volume at the health facility. The study was conducted at 245 public and private HIV clinics in the 5 regions of Uganda. Women were included in the survey if they were HIV positive and receiving HIV care and treatment in the selected health facilities, aged 15–49 years and sexually active within the past 12 months. Women who did not give consent were excluded from the study.

### Assessment of intimate partner violence

For the purposes of this study, the definition of intimate partner violence was restricted to experiencing any physical or sexual violence. A questionnaire was used to assess women’s experience of any form of physical or sexual violence by their partner. Participants were asked the question “Does your current husband or partner ever hit, slap, kick or do anything else to hurt you physically?” Women who responded “yes” to this question were classified as having ever experienced any form of physical violence. In addition, women were asked, “Does your current husband or partner physically force you to have intercourse or perform any other sexual acts against your will?” Women who responded “yes” to this question were classified as having ever experienced any form of sexual violence. Participants were also asked about possible predictors such as their partner’s age, HIV status disclosure to sexual partner (and whether or not both partners had mutually shared their HIV results), relationship status and HIV status of partner, HIV treatment status (ART versus non-ART and duration on treatment) and other demographic information such as age, highest level of education attained, marital status and ownership of household possessions such as a mobile phone, television set or radio. The questionnaire was pre-tested to check the suitability of various aspects such as the clarity of the translation, skip patterns and filtering questions. In order to ensure internal validity of the questionnaire, continuous roles plays among the 5 research assistants from each of the 5 regional teams were conducted for two days after training to ensure a thorough critic of the flow of the tools. This approach appeared more rewarding compared with the conventional approach of field pretesting of study tools as the research assistants had more time to interact with tools and as such became more familiar with them. There was sufficient time for comparison of scenarios, discussion and revision of the tools. Each interview was carried out by a trained interviewer after obtaining informed consent from the interviewee. The nature of the interview process was one-to-one and all responses given by participants were made anonymous to protect the confidentiality of the data collected. The questionnaire also emphasized the anonymous nature of the responses to allow for the disclosure of sensitive information like the partners HIV status without fear of any consequences. The questionnaire was translated into five major languages; Luganda for Central region and Kampala, Runyakitara for Western region, Ateso and Lusoga for the Eastern region and Luo for the Northern region. Data were collected between September and November 2016.

### Statistical analysis

We computed the number of women who had experienced any form of intimate partner violence by adding the number of those that had experienced any physical violence to those that had experienced any sexual violence. This was presented as a percentage of all the participants in the survey.

To determine the factors associated with experiencing intimate partner violence, we used prevalence risk ratios (PRR) as the measure of association. PRRs were obtained via a modified Poisson regression model with robust standard errors. This statistical technique was preferred over ordinary logistic regression which overestimates the prevalence ratios [15] and poorly estimates the standard errors of the estimated risk ratios when the outcome of interest is not rare [16, 17]. The independent variables assessed were those related to the woman’s HIV status such as the duration of time they have been on anti-retroviral treatment (ART), HIV status disclosure between the woman and their partner, HIV status of the partner, relationship variables such as the age of the partner and spousal age difference and demographic variables such as marital status, highest level of education attained, number of biological children and the social economic status (wealth quintile). Initially, each of the independent variables was run against the dependent variable in a bivariate model. Thereafter, all variables with a *p*-value < 0.1 in the bivariate model were included in a multivariable analysis. When running a multiple regression model, two or more variables may be highly correlated in a phenomenon referred to as multicollinearity. This leads to wrong estimates of the standard errors of the correlated variables thus compromising the statistical significance of including these variables in a model [18]. We therefore used the variance inflation factor to assess for multicollinearity between independent variables such as the partner’s age and spousal age difference and HIV status disclosure between the woman and their partner and HIV status of the partner. A variance inflation factor less than 10 showed that there was no multicollinearity between the variables investigated [19]. All analyses were conducted using STATA version 13.

## Results

A total of 5198 HIV positive women participated in the survey. 2441 (47.0%) were aged 20–39 years; 3079 (59.2%) were married; 2924 (56.3%) had obtained primary level education and almost all; 5022 (96.9%) were on antiretroviral treatment (Table 1).

**Table 1:** Characteristics of participants in the survey

Characteristic	Number	%
Age		
15–19	103	2.0
20–24	657	12.6
25–29	1147	22.1
30–34	1294	24.9
35–39	960	18.5
≥ 40	1037	20.0
Marital status		
Never married	107	2.1
In relationship but not married	1371	26.4
Married	3079	59.2
Divorced/ Separated/ Widowed	641	12.3
Highest level of education		
No education	726	14.0
Primary	2924	56.3
Secondary	1381	26.6
Tertiary/ University or higher	156	3.0
Missing	11	0.2
Region of Residence		
Kampala	1048	20.2
Central	1032	19.9
Eastern	1034	19.9
Western	1039	20.0
Northern	1045	20.1
Owns a radio		
No	1822	35.1
Yes	3376	64.9
Owns a mobile phone		
No	991	19.1
Yes	4207	80.9
Owns a bicycle		
No	3583	68.9
Yes	1615	31.1
Number of biological children		
0	1342	25.8
1–2	1440	27.7
3–4	1435	27.6
≥ 5	981	18.9
On Antiretroviral Therapy (ART)		
No	161	3.1
Yes	5022	96.9
Health facility level		
Hospital	1556	29.9
Health Center IV	1540	29.6
Health Center III	1542	29.7
Health Center II	416	8.0
Private health unit	112	2.2
Other	32	0.6

Of the 5198 women that participated in the survey, 1664 (32.1) reported to have experienced physical violence where by the husband or partner ever hit, slapped, kicked or did something to hurt the woman physically (Table 2). In addition, 1466 (28.3%) reported to have experienced sexual violence where the husband or partner ever physically forced them to have intercourse or perform any other sexual acts against their will. A total of 2290 (44.2%) women reported to have experienced. Physical or sexual violence. Among those who were married, 1126 (36.6%) had ever experienced physical violence, 916 (29.7%) had ever experienced sexual violence and 1491 (48.4%) had experienced some form of intimate partner violence.

**Table 2:** Participants in the survey who have experienced different forms of Intimate Partner Violence

		Physical Violence	Sexual violence	Physical or Sexual violence
Characteristic	-n-	-n-(%)	-n-(%	-n-(%)
Total	5198	1664 (32.1)	1466 (28.3)	2290 (44.2)
Age				
15–19	103	24 (23.3)	16 (15.5)	31 (30.1)
20–24	657	190 (28.9)	165 (25.1)	267 (40.6)
25–29	1147	367 (32.0)	312 (27.2)	500 (43.6)
30–34	1294	450 (34.8)	383 (29.6)	598 (46.2)
35–39	960	307 (32.0)	290 (30.2)	443 (46.1)
≥ 40	1037	326 (31.4)	300 (28.9)	451 (43.5)
Partner’s Age				
15–24	203	57 (28.1)	40 (19.7)	74 (36.5)
25–34	1468	466 (31.7)	387 (26.4)	632 (43.1)
35–44	1908	630 (33.0)	581 (30.5)	881 (46.2)
45–54	1171	374 (31.9)	343 (29.3)	516 (44.1)
≥ 55	448	137 (30.6)	115 (25.7)	187 (41.7)
Marital status				
Never married	107	17 (15.9)	21 (19.6)	26 (24.3)
In relationship but not married	1371	372 (27.1)	359 (26.2)	533 (38.9)
Married	3079	1126 (36.6)	916 (29.7)	1491 (48.4)
Divorced/ Separated/ Widowed	641	149 (23.2)	170 (26.5)	240 (37.4)
Highest level of education				
No education	723	241 (33.3)	222 (30.7)	323 (44.8)
Primary	2910	1004 (34.5)	867 (29.8)	1361 (46.8)
Secondary	1372	391 (28.5)	350 (25.5)	560 (40.8)
Tertiary/ University or higher	156	22 (14.1)	27 (17.3)	40 (25.6)
Region of Residence				
Kampala	1048	323 (30.8)	277 (26.4)	451 (43.0)
Central	1032	388 (37.6)	315 (30.5)	492 (47.7)
Eastern	1034	331 (32.0)	270 (26.1)	458 (44.3)
Western	1039	238 (22.9)	268 (25.8)	387 (37.2)
Northern	1045	384 (36.7)	336 (32.2)	502 (48.0)
Number of biological children				
0	1342	301 (22.4)	325 (24.2)	470 (35.0)
1–2	1440	451 (31.3)	371 (25.8)	612 (42.5)
3–4	1435	526 (36.7)	461 (32.1)	706 (49.2)
≥ 5	981	386 (39.3)	309 (31.5)	502 (51.2)
On Antiretroviral Therapy (ART)				
No	161	45 (28.0)	41 (25.5)	63 (39.1)
Yes	5022	1616 (32.2)	1423 (28.3)	2223 (44.3)
Wealth quintile				
Lowest	1054	359 (34.1)	322 (30.6)	480 (45.5)
Second	1026	351 (34.2)	286 (27.9)	474 (46.2)
Middle	1041	336 (32.3)	323 (31.0)	473 (45.4)
Fourth	1039	309 (29.7)	279 (26.9)	438 (42.2)
Highest	1038	309 (29.8)	256 (24.7)	425 (40.9)
Nature of relationship				
Monogamous	1173	460 (39.2)	392 (33.4)	606 (51.7)
Polygamous	1858	649 (34.9)	510 (27.4)	862 (46.4)
Duration on ART (years)				
≤ 1	1131	350 (30.9)	297 (26.3)	483 (42.7)
2	738	266 (36.0)	228 (30.9)	354 (48.0)
3	720	238 (33.1)	198 (27.5)	322 (44.7)
4	555	187 (33.7)	168 (30.3)	254 (45.8)
≥ 5	2041	623 (30.5)	575 (28.2)	877 (43.0)
HIV status disclosure				
Both know each other’s status	1631	518 (31.2)	441 (27.0)	714 (43.8)
Status not mutually known	1862	578 (31.0)	536 (28.8)	819 (44.0)
HIV status with partner				
Both positive	1887	653 (34.6)	568 (30.1)	885 (46.9)
Woman positive and partner negative	581	169 (29.1)	141 (24.3)	235 (40.4)

The multivariable analysis revealed that compared with women in relationships where the woman and her partner were of the same age, women in relationships where the partner was younger were more likely to experience intimate partner violence PRR = 1.43, 95% CI: 1.14–1.79, as were women in relationships where the partner was < 10 years older PRR = 1.20, 95% CI: 1.00–1.43, and women in relationships where the partner was ≥10 years older PRR = 1.31 95% CI: 1.05–1.64 (Table 3). Compared with women who did not have any biological children, women with 3–4 biological children were more likely to experience intimate partner violence PRR = 1.27 95% CI: 1.00–1.59 as were those with 5 or more biological children PRR = 1.34 95% CI: 1.06–1.71. Compared with women in relationships where both the woman and her partner are HIV positive, women in relationships where the woman was HIV positive but the partner was HIV negative were less likely to experience intimate partner violence PRR = 0.87 95% CI: 0.78–0.98.

**Table 3:** Bivariate and multivariate analyses showing Prevalence Risk Ratios (PRR) of experiencing any form of Intimate Partner Violence among HIV Positive Women in care in Uganda, 2016

Characteristic	-n-	Bivariate		Multivariate	
		PRR [95%CI]	p-value	PRR [95%CI]	p-value
Age					
15–19	103	1.0		1.0	
20–24	657	1.35 [0.99–1.84]	0.055	1.24 [0.73–2.11]	0.417
25–29	1147	1.45 [1.07–1.96]	0.015	1.32 [0.77–2.24]	0.309
30–34	1294	1.54 [1.14–2.07]	0.005*	1.42 [0.83–2.44]	0.206
35–39	960	1.54 [1.14–2.08]	0.005*	1.48 [0.85–2.59]	0.167
≥ 40	1037	1.45 [1.07–1.96]	0.016*	1.44 [0.81–2.57]	0.217
Partner’s age					
15–24	203	1.0		1.0	
25–34	1468	1.18 [0.98–1.43]	0.084	0.93 [0.72–1.28]	0.631
35–44	1908	1.27 [1.05–1.53]	0.013*	0.87 [0.62–1.20]	0.388
45–54	1171	1.21 [0.99–1.47]	0.053	0.80 [0.55–1.16]	0.241
≥ 55	448	1.15 [0.93–1.42]	0.189	0.75 [0.47–1.19]	0.223
Spousal age difference					
Same age	545	1.0		1.0	
Partner younger	379	1.14 [0.98–1.32]	0.083	1.43 [1.14–1.79]	0.002*
Partner < 10 years older	2735	1.07 [0.96–1.20]	0.204	1.20 [1.00–1.43]	0.045*
Partner ≥10 years older	1324	1.11 [0.99–1.25]	0.075	1.31 [1.05–1.64]	0.017*
Marital status					
Married	3079	1.0		1.0	
In relationship but not married	1371	0.80 [0.75–0.87]	< 0.05*	0.96 [0.85–1.08]	0.483
Never married	107	0.51 [0.36–0.71]	< 0.05*	0.97 [0.55–1.71]	0.924
Divorced/ Separated/ Widowed	641	0.77 [0.70–0.86]	< 0.05*	1.07 [0.82–1.40]	0.621
Highest level of education					
No education	723	1.0		1.0	
Primary	2910	1.05 [0.96–1.14]	0.333	1.08 [0.95–1.24]	0.220
Secondary	1372	0.91 [0.82–1.01]	0.081	0.98 [0.84–1.15]	0.782
Tertiary/ University or higher	156	0.57 [0.43–0.76]	< 0.05*	0.76 [0.52–1.11]	0.162
Number of biological children					
0	1342	1.0		1.0	
1–2	1440	1.22 [1.11–1.34]	< 0.05*	1.20 [0.95–1.51]	0.123
3–4	1435	1.40 [1.28–1.54]	< 0.05*	1.27 [1.00–1.59]	0.045*
≥ 5	981	1.46 [1.33–1.61]	< 0.05*	1.34 [1.06–1.71]	0.017*
Wealth quintile					
Lowest	1054	1.0		1.0	
Second	1026	1.02 [0.92–1.11]	0.747	0.93 [0.82–1.05]	0.241
Middle	1041	0.99 [0.91–1.10]	0.994	0.96 [0.84–1.09]	0.498
Fourth	1039	0.93 [0.84–1.02]	0.138	0.96 [0.84–1.10]	0.589
Highest	1038	0.90 [0.82–0.99]	0.036*	0.90 [0.77–1.05]	0.181
Duration on ART (years)					
≤ 1	1131	1.0		1.0	
2	738	1.12 [1.02–1.24]	0.024*	1.10 [0.94–1.28]	0.229
3	720	1.05 [0.94–1.16]	0.392	1.04 [0.89–1.21]	0.641
4	555	1.07 [0.96–1.20]	0.230	1.07 [0.90–1.26]	0.454
≥ 5	2041	1.01 [0.93–1.09]	0.886	1.02 [0.89–1.17]	0.904
HIV status disclosure					
Both know each other’s status	1631	1.0		1.0	
Status not mutually known	1862	1.00 [0.93–1.08]	0.902	Omitted	
HIV status with partner					
Both positive	1887	1.0		1.0	
Woman positive and partner negative	581	0.86 [0.77–0.96]	0.008*	0.87 [0.78–0.98]	0.017*

*Indicates statistical significance

## Discussion

This analysis has revealed that more than 4 in every 10 HIV infected women in care in Uganda (44.2%) experience physical and/ or sexual violence perpetrated by their intimate partner. The prevalence of physical intimate partner violence was higher (32.1%) than that of sexual intimate partner violence (28.3%). These findings are comparable to those from the 2016 Uganda Demographic Health Survey that showed that 44 and 25% of women aged 15–49 years in the general population had experienced spousal physical and spousal sexual violence respectively [5]. Compared with findings from our study, a follow up study conducted in Mbarara District in western Uganda established that the prevalence of current intimate partner violence among women living with HIV ranged from 3 to 11% between 2011 and 2015 [20]. The lower prevalence could be explained by factors such as culture of the people that are specific to this region in Uganda. A study conducted in Nigeria found that 23.6% of women living with HIV had experienced violence by their intimate partner [21] which is lower compared to the findings in our study. In Nepal, 93% of women living with HIV reported to experience violence although 45% reported their partner as being the perpetrator of this violence [22]. In the US, albeit in a different sociocultural context, one study reported that 35% of women living with HIV experienced sexual violence [23] and another reported that 62% experienced physical and/ or sexual violence [24]. Both studies however did not specify the perpetrators of the violence.

Among several factors, violence perpetrated by a male partner within an intimate relationship has been attributed to the desire to dominate and control women when there is conflict and particularly in patriarchal societies where the violence is endorsed by both men and women [4, 25, 26]. This desire to have power over and control one’s partner might be the explanation to our observation that compared with women in relationships where the woman and her partner were of the same age, women in relationships where the woman and her partner were of different age were more likely to experience intimate partner violence. Targeted interventions for both women and men should be developed to promote alternative conflict resolution strategies such as communication and negotiation, elevate the societal perception of women and promote their social and economic empowerment [25]. Given the high prevalence of IPV, care and treatment programs for women living with HIV should involve screening of women for IPV so that the appropriate psychosocial and medical attention can be provided to them.

Compared with women who did not have any biological children, women with 3–4 biological children and those with ≥5 biological children were more likely to report experiencing any form of intimate partner violence. Several studies have consistently reported that the number of children in the home is a significant risk factor for IPV because having a child could amplify stress factors such as financial responsibilities that accompany having one [27–30]. In homes where IPV was already existing, having a child could put a strain on the relationship which increases the possibility of IPV [30]. Noteworthy though is that these studies were not conducted among women living HIV and were unclear about the biological relationship between the children and the woman experiencing the violence. We would like to speculate however that the number of biological children a woman has might have been a proxy indicator for the length of the union between the partners and the observed association between the number of biological children a woman has and experience of IPV probably reflects the longer period of the union and increased likelihood of conflict and violence. A multi country study established that across several sites, women who had been in unions for shorter periods of time reported fewer incidents of intimate partner violence [31].

HIV positive women in sero-discordant relationships were less likely to experience any form of IPV compared with those in sero-concordant relationships. Compared with HIV negative women, women who are HIV positive have been shown to be at significant risk of experiencing intimate partner violence [32, 33]. Some studies have reported that the revelation of HIV status sero-discordance in an intimate relationship may introduce or escalate the level of violence due to fear of HIV transmission and disagreements about condom use, apportioning of blame for the source of the HIV infection, suspicions of infidelity and subsequent alcohol abuse [34]. When HIV positive women in sero-discordant relationships are compared with women in sero-concordant relationships, our findings are consistent with the results of a prospective study of 3408 HIV sero-discordant couples which established that the frequency of IPV reduced over the 2 year period of follow up and was similar or lower compared with the frequency in the general population [35]. The study conducted in western Uganda among HIV positive women also showed that women reported progressively lower cases of current intimate partner violence over the 5 year study period [20]. In addition, the lower prevalence of IPV in sero-discordant relationships might reflect the extra support and attention in terms of counseling these couples receive in anticipation of the violence compared with their positive concordant counterparts in a setting like Uganda where the prevalence of IPV is already high. Awareness about IPV and acknowledgment of its likelihood should be included into post HIV testing counseling services to minimize the risk of subsequent conflict among HIV sero-discordant couples so that these relationships can quickly transition to a state of acceptance and mutual support.

This study is not without limitations. The analysis was based on data from a survey the primary objective of which was to establish the unmet need for family planning among HIV+ women. Other variables that have been shown to be predictors of intimate partner violence such as life time exposure to violence and alcohol and drug use [36] were therefore not assessed. The experience of intimate partner violence which was our main outcome variable was based on self-reports which is a source of information bias. In this paper, intimate partner violence was narrowly defined as physical and sexual violence excluding emotional/ psychological violence and controlling behavior both of which were not assessed. Thus, there was an underestimate of the prevalence of intimate partner violence which biased our associations towards the null hypothesis.

## Conclusions

There is a high level of violence against HIV positive women in care with more than 4 in every 10 reporting to experience violence perpetrated by their intimate partners. Health care workers should screen women for intimate partner violence using standard approaches and offer or recommend the appropriate psychosocial or medical assistance. Experiencing intimate partner violence was associated with circumstances related to the relationship between the woman and her intimate partner. These data can inform the design of interventions for integrating strategies to curb intimate partner violence into HIV programming and identifying HIV positive women in care at risk of experiencing intimate partner violence.

## Data Availability

The data that were used in the analyses for the manuscript are not publicly available. They could however be availed upon reasonable request by writing an email to the corresponding author.

## Abbreviations

ART: Anti-Retroviral Therapy
HC: Health Center
HIV: Human Immunodeficiency Virus
IPV: Intimate Partner Violence
PRR: Prevalence Risk Ratio
WHO: World Health Organization
